# Network Pharmacology Study on the Pharmacological Mechanism of Cinobufotalin Injection against Lung Cancer

**DOI:** 10.1155/2020/1246742

**Published:** 2020-02-17

**Authors:** Yun Mao, Xi Peng, Peng Xue, Dianrong Lu, Linlu Li, Shijie Zhu

**Affiliations:** ^1^Graduate School, Beijing University of Chinese Medicine, Beijing 100029, China; ^2^Department of Oncology, Wangjing Hospital, China Academy of Chinese Medical Sciences, Beijing 100102, China; ^3^Department of Cardiology, Hunan Provincial People's Hospital, Changsha 410000, China

## Abstract

Cinobufotalin injection, extracted from the skin of Chinese giant salamander or black sable, has good clinical effect against lung cancer. However, owing to its complex composition, the pharmacological mechanism of cinobufotalin injection has not been fully clarified. This study aimed to explore the mechanism of action of cinobufotalin injection against lung cancer using network pharmacology and bioinformatics. Compounds of cinobufotalin injection were determined by literature retrieval, and potential therapeutic targets of cinobufotalin injection were screened from Swiss Target Prediction and STITCH databases. Lung-cancer-related genes were summarized from GeneCards, OMIM, and DrugBank databases. The pharmacological mechanism of cinobufotalin injection against lung cancer was determined by enrichment analysis of gene ontology and Kyoto Encyclopedia of Genes and Genomes, and protein-protein interaction network was constructed. We identified 23 compounds and 506 potential therapeutic targets of cinobufotalin injection, as well as 70 genes as potential therapeutic targets of cinobufotalin injection in lung cancer by molecular docking. The antilung cancer effect of cinobufotalin injection was shown to involve cell cycle, cell proliferation, antiangiogenesis effect, and immune inflammation pathways, such as PI3K-Akt, VEGF, and the Toll-like receptor signaling pathway. In network analysis, the hub targets of cinobufotalin injection against lung cancer were identified as VEGFA, EGFR, CCND1, CASP3, and AKT1. A network diagram of “drug-compounds-target-pathway” was constructed through network pharmacology to elucidate the pharmacological mechanism of the antilung cancer effect of cinobufotalin injection, which is conducive to guiding clinical medication.

## 1. Introduction

Lung cancer is the world's most malignant tumor with high morbidity and mortality, and it includes non-small-cell lung cancer (NSCLC) and small-cell lung cancer [1]. The latest research showed that the 5-year survival rate of lung cancer in the United States is approximately 19% [2]. The main treatments for lung cancer include surgery, chemotherapy, radiotherapy, targeted therapy, and immunotherapy. In recent years, breakthroughs have been made in immunotherapy and targeted therapy for lung cancer. In contrast to chemotherapy, Keytruda® (pembrolizumab) significantly prolongs patient survival in patients with lung cancer who have high expression level of PD-L1 (PD-L1 ≥ 50%) and no EGFR or ALK mutations [3]. The US Food and Drug Administration (FDA) has approved pembrolizumab for the first-line treatment of lung cancer. However, there are still problems such as easy recurrence and obvious adverse reactions during the treatment of lung cancer; thus, it is necessary to actively explore new treatment methods.

Traditional Chinese medicine (TCM) has been widely used in the prevention and treatment of diseases in East Asia (China, Japan, and Korea) for more than two thousand years. Chinese herbal medicine, including botanical, animal, and mineral medicines, is an important part of TCM. In recent years, TCM has received increasing attention. For example, Professor Zheng Yengqin of Yale University used Huangqin Decoction to treat malignant tumors [4]. In addition, the 72nd World Health Assembly reviewed and approved the 11th International Classification of Diseases (ICD-11), which was first incorporated into ancient Chinese traditional medicine. TCM can directly inhibit the growth and proliferation of malignant tumors; for example, arsenic trioxide can be used to treat acute promyelocytic leukemia. It can also be used for adjuvant treatment of surgery, chemotherapy, radiotherapy, etc., which can increase the curative effect and reduce adverse reactions [5].

Cinobufotalin injection, a water-soluble preparation extracted from Chinese giant salamander or black sable, has been approved by the China Food and Drug Administration (ISO9002) for the treatment of malignant tumors [6, 7]. Jiang et al. [8] carried out maintenance therapy in 64 patients with NSCLC who did not progress after first-line chemotherapy. The control group received maintenance therapy with chemotherapy drugs, whereas the experimental group received maintenance therapy with cinobufotalin injection and Chinese herbal decoction. The results showed that there was no difference in median overall survival between the experimental and control groups (*P* = 0.601). Cinobufotalin injection increased the 1-year survival rate (78.1% vs. 53.1%, *P* = 0.035) and improved the quality of life of patients. A meta-analysis of 1,142 NSCLC patients showed that cinobufotalin injection exerts antitumor effect, thus improving disease control rate and patients' quality of life and reducing the incidence of myelosuppression [9]. However, modern research mostly focuses on a single compound of cinobufacin injection, which does not reflect the antitumor mechanism of the multicompound, multitarget, and multichannel system of Chinese herbal medicine. In recent years, the rise and vigorous development of network pharmacology has provided new ideas and new solutions for studying the mechanism of action of Chinese herbal medicine. From the perspective of network pharmacology, this study explored the target and key signaling pathway of cinobufacin injection for lung cancer, providing potential strategies for the treatment of lung cancer.

## 2. Materials and Methods

### 2.1. Compounds of Cinobufotalin Injection

We searched the literature on compounds of cinobufotalin injection using PubMed, Web of Science, and China National Knowledge Infrastructure. The keywords used were “dry scalp,” “cinobufotalin injection,” and “Huachansu injection.” The literature search screened 26 compounds. Biomolecular activity data were obtained from PubChem (https://pubchem.ncbi.nlm.nih.gov) [10], which is a database of chemical modules supported by the National Institutes of Health (NIH) and maintained by the National Center for Biotechnology Information (NCBI). All screened compounds were input into the PubChem database, whereas compounds with duplicated data and without structural information were removed. We obtained the canonical Simplified Molecular Input Line Entry Specification (SMILES) and structural information of 23 compounds [7, 11–13]. The specific results are shown in [Supplementary-material supplementary-material-1]. The obtained compounds included bufalin, bufotenidine, arenobufagin, and resibufogenin.

### 2.2. Prediction of Targets of Cinobufotalin Injection

The Swiss Target Prediction (http://www.swisstargetprediction.ch/) [14] and the Search Tool for Interacting Chemicals (STITCH) (http://stitch.embl.de/) [15] databases are widely used in the research of Chinese medicine mechanisms and potential therapeutic targets. The 23 compounds of cinobufotalin injection have SMILES information, which can be used to predict potential targets according to Swiss Target Prediction and STITCH databases. Potential therapeutic targets were predicted by examining the similarity between the 2D and 3D structures of the compounds. The screening condition was limited to “Homo sapiens,” and high probability targets were selected after duplicate contents were eliminated.

### 2.3. Prediction of Targets for Lung Cancer

We used online tools such as GeneCards (http://www.genecards.org/) [16], Online Mendelian Inheritance in Man (OMIM, http://www.omim.org/) [17], and DrugBank (http://www.drugbank.ca/) [18] to find potential therapeutic targets for lung cancer. The search conditions were set to “gene” and “Homo sapiens,” and the authenticity of the related genes was determined by literature search. To improve the accuracy of the forecast, in the DrugBank database, we only selected the target of drug action approved by FDA [18].

### 2.4. Functional Enrichment Analysis

The Database for Annotation, Visualization, and Integrated Discovery (DAVID) (https://david.ncifcrf.gov/), a widely used web-based genomic functional annotation tool, was used for functional analysis of the potential therapeutic targets of cinobufotalin injection [19]. Gene ontology (GO) analysis and the Kyoto Encyclopedia of Genes and Genomics (KEGG) pathway analysis in the DAVID online tool were used for exploring gene function.

### 2.5. Protein-Protein Interaction (PPI) Network Construction

We constructed a PPI network to elucidate the molecular mechanisms of the antilung cancer effects of cinobufotalin injection by using the Cytoscape software (version 3.6.0; http://www.cytoscape.org) [20] and the STRING database (version 10.0, http://www.string-db.org/) with a required confidence >0.4 [21]. Subsequently, we confirmed the hub genes in the PPI network using the molecular complex detection (MCODE) plugin of the Cytoscape software. The screening thresholds were “degree cutoff = 2,” “node score cutoff = 0.2,” “k-core = 2,” and “max·depth = 100.”

### 2.6. Relationship between Hub Genes and Prognosis

The hub genes related to the pharmacological effects of cinobufotalin injection on lung cancer were identified by constructing a complex and multicenter network map. The relationship between the expression level of hub genes and prognosis was analyzed by using Kaplan-Meier Online website (http://kmplot.com/analysis/), which contains data on survival prognosis of lung cancer in The Cancer Genome Atlas (TCGA) and Gene Expression Omnibus (GEO) databases [22]. We used data from the Kaplan-Meier Online website for lung adenocarcinoma and lung squamous cell carcinoma to analyze the relationship between VEGFA, EGFR, CCND1, CASP3, and AKT1 and patient prognosis.

## 3. Results

### 3.1. Potential Targets of Cinobufotalin Injection

**Figure 1:**
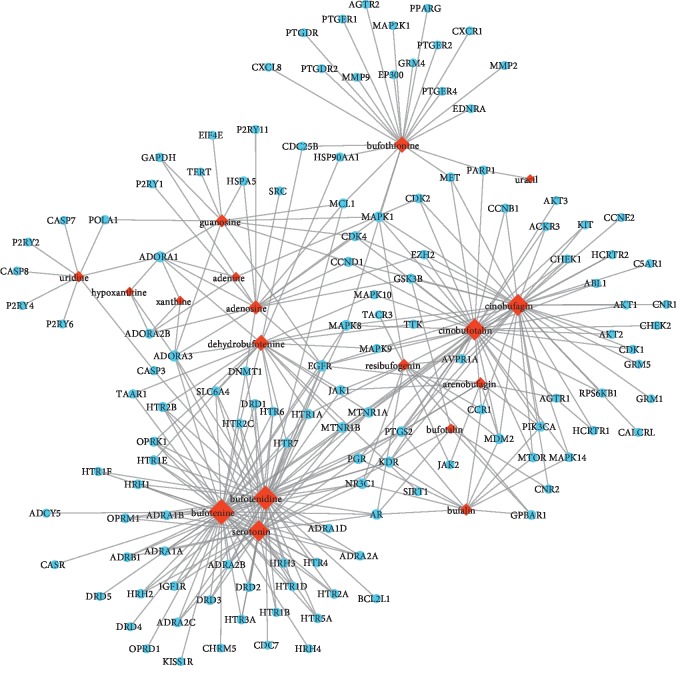
PPI network of “compound-target” in cinobufotalin injection. The bigger the graph, the bigger the degree. Rhomboids represent compounds and circles represent potential therapeutic targets. PPI: protein‐protein interaction.

In total, 506 potential targets of 23 compounds were identified using Swiss Target Prediction and STITCH databases. A visual PPI network of the 506 potential targets was subsequently constructed using the Cytoscape software. When the interaction score was greater than 0.4, a complex network composed of multiple nodes and edges was constructed after isolated and partially loosely connected gene nodes were deleted. The generated visual PPI network of potential therapeutic targets for cinobufotalin injection contained 503 nodes and 7191 edges. In the PPI network, there were 132 genes whose degree was greater than or equal to 10, as determined by MCODE plugin, and these genes were regulated by 18 compounds (Figure 1). Among the 132 genes, the 10 potential therapeutic targets with the highest degree of nodes were considered as hub genes. These hub genes included AKT1 (degree = 183), GAPDH (degree = 180), VEGFA (degree = 131), SRC (degree = 128), EGFR (degree = 125), MAPK1 (degree = 123), CASP3 (degree = 120), HSP90AA1 (degree = 116), CXCL8 (degree = 115), and CCND1 (degree = 108).

### 3.2. Functional Enrichment Analysis of Cinobufotalin Injection

**Figure 2:**
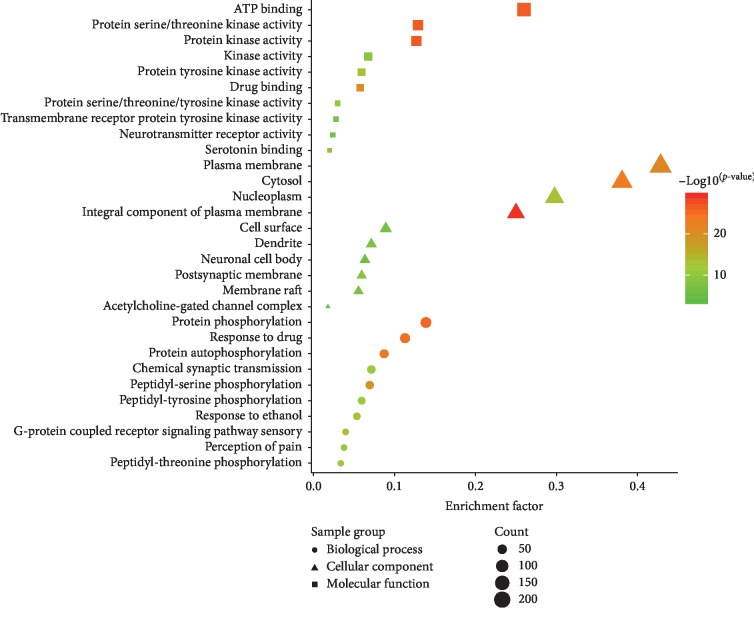
GO analysis of potential target genes of cinobufotalin injection. GO: gene ontology.

**Figure 3:**
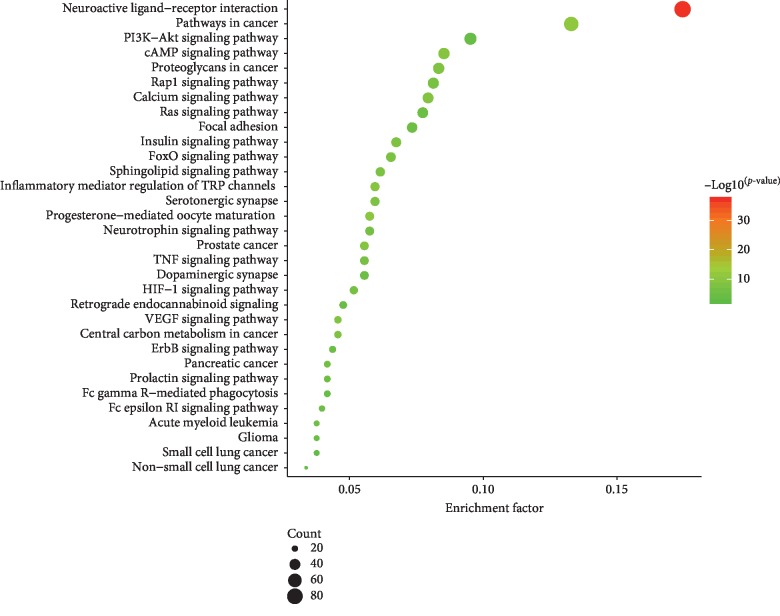
KEGG analysis of potential target genes of cinobufotalin injection. KEGG: Kyoto Encyclopedia of Genes and Genomes.

The biological classification of 506 potential therapeutic targets of cinobufotalin injection was analyzed utilizing the functional enrichment analysis of the DAVID website. This results showed 1083 enriched GO functions, which mainly involved biological processes (756 items), cell components (99 items), and molecular functions (228 items). We set the condition of statistical analysis as the false discovery rate (FDR) corrected *P* < 0.05 and screened the top 10 entries with significant enrichment in biological processes, cell components, and molecular functions, as shown in Figure 2. For biological processes, potential therapeutic targets of cinobufotalin injection were significantly enriched in “protein phosphorylation,” “response to drug,” and “protein autophosphorylation.” For cell components, mainly, “integral component of plasma membrane,” “cytosol,” and “membrane” were enriched. In addition, 10 enriched items belonged to molecular functions, including “protein kinase activity,” “ATP binding,” and “drug binding.” Subsequently, 506 genes were enriched in 138 pathways, as determined by KEGG analysis. The FDR corrected *P* < 0.05 was used as an enrichment screening criterion, and the top 32 enriched functional clusters of potential therapeutic targets were obtained (Figure 3), including “non-small cell lung cancer” (17 genes), “small cell lung cancer” (19 genes), “TNF signaling pathway” (28 genes), “HIF-1 signaling pathway” (26 genes), “PI3K-Akt signaling pathway” (48 genes), and “T cell receptor signaling pathway” (22 genes).

### 3.3. Potential Targets of Lung Cancer

**Figure 4:**
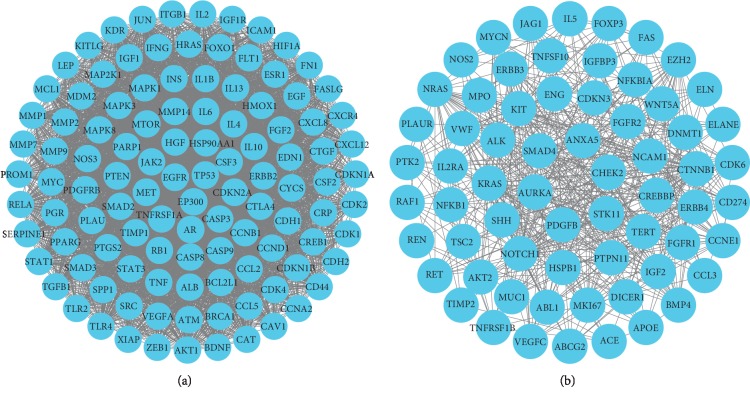
Top two modules of lung cancer from the protein-protein interaction network analysis. (a) is Module 1 and (b) is Module 2.

A total of 461 lung cancer targets were retrieved from the GeneCards, OMIM, and DrugBank databases and visually analyzed by the Cytoscape software. A PPI network containing 377 nodes and 14204 edges was constructed based on 461 lung cancer genes. We used MCODE plugin to analyze the core genetic association of the network, with 176 genes with a genomic degree ≥15. In total, 176 genes belonged to Module 1 and Module 2, respectively, as shown in Figure 4. In addition, TP53, EGFR, AKT1, MYC, VEGFA, INS, IL6, HRAS, PTEN, and CCND1 were shown as the most potential therapeutic targets of lung cancer, and they were considered as hub genes. The 461 lung cancer targets were analyzed by GO and KEGG functional enrichment analyses. GO analysis revealed that the FDR value of 334 items was less than 0.05, including apoptosis, cell cycle, and angiogenesis ([Supplementary-material supplementary-material-1]). KEGG analysis showed that the occurrence and development of lung cancer were related to multiple signaling pathways, such as the TP53, PI3K-Akt, and MAPK signaling pathways ([Supplementary-material supplementary-material-1]).

### 3.4. Pharmacological Mechanism of Cinobufotalin Injection against Lung Cancer

**Figure 5:**
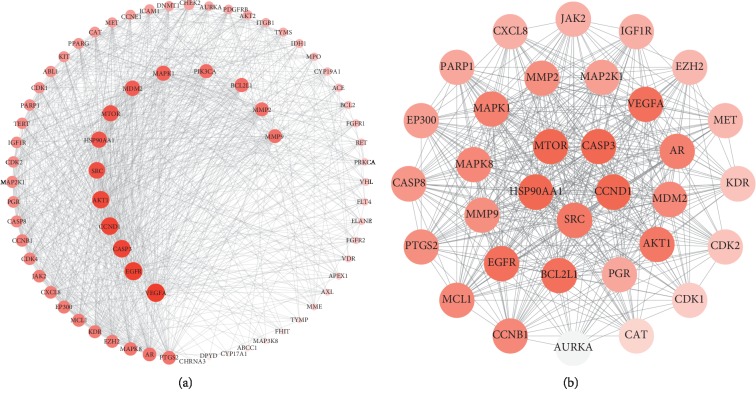
(a) PPI network of potential target genes of cinobufotalin injection for lung cancer. The larger the circle, the greater the degree. (b) Top one module of the potential target genes of cinobufotalin injection for lung cancer. The darker the color, the greater the degree. PPI: protein‐protein interaction.

We used Venny2.1 (https://bioinfogp.cnb.csi) to find overlapping genes between potential therapeutic targets of cinobufotalin injection and lung cancer-related targets, and a complex, multicenter, multilateral interaction network map with 70 nodes and 965 edges was constructed (Figure 5(a)). In this study, we used the MCODE plugin of the Cytoscape software to analyze the correlation between 70 genes and found that 33 genes belonged to the hub genes with a degree of higher than 10. Among which, the 10 genes with the highest node degree were as follows: VEGFA, EGFR, CCND1, CASP3, AKT1, SRC, MTOR, HSP90AA1, MDM2, and MAPK1 (Figure 5(b)).

**Figure 6:**
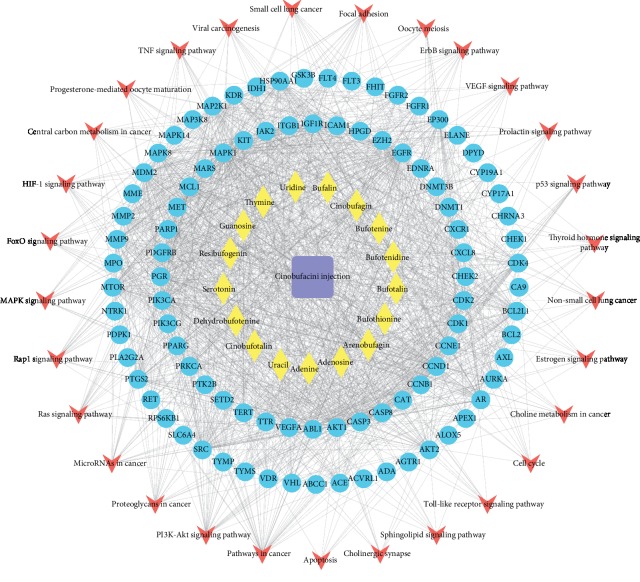
PPI network of “drug-compound-target-pathway” of cinobufotalin injection for lung cancer. The square represents cinobufotalin injection, the diamond represents drug composition, the circle represents the target of cinobufotalin injection for lung cancer, and the concave quadrangle represents the signaling pathway. PPI: protein‐protein interaction.

To further analyze the pharmacological mechanism of cinobufotalin injection against lung cancer, GO and KEGG analyses of 70 overlapping genes were performed. GO analysis revealed 69 items with FDR corrected *P* < 0.05. The top 20 GO entries included “protein tyrosine kinase activity,” “peptidyl-tyrosine phosphorylation,” and “ATP binding” (Table 1). Subsequently, we used KEGG analysis to identify enriched signaling pathways. The results showed, mainly, pathways involved in tumor cell proliferation, apoptosis, and immunomodulation, such as “p53 signaling pathway,” “PI3K-Akt signaling pathway,” “HIF-1 signaling pathway,” and “cell cycle,” as shown in Table 2. In addition, a network of “drug-compound-gene-pathway” was constructed by the Cytoscape software to fully reflect the pharmacological mechanism of cinobufotalin injection against lung cancer (Figure 6).

### 3.5. Effect of Key Genes on Median Survival of Lung Cancer Patients

**Figure 7:**
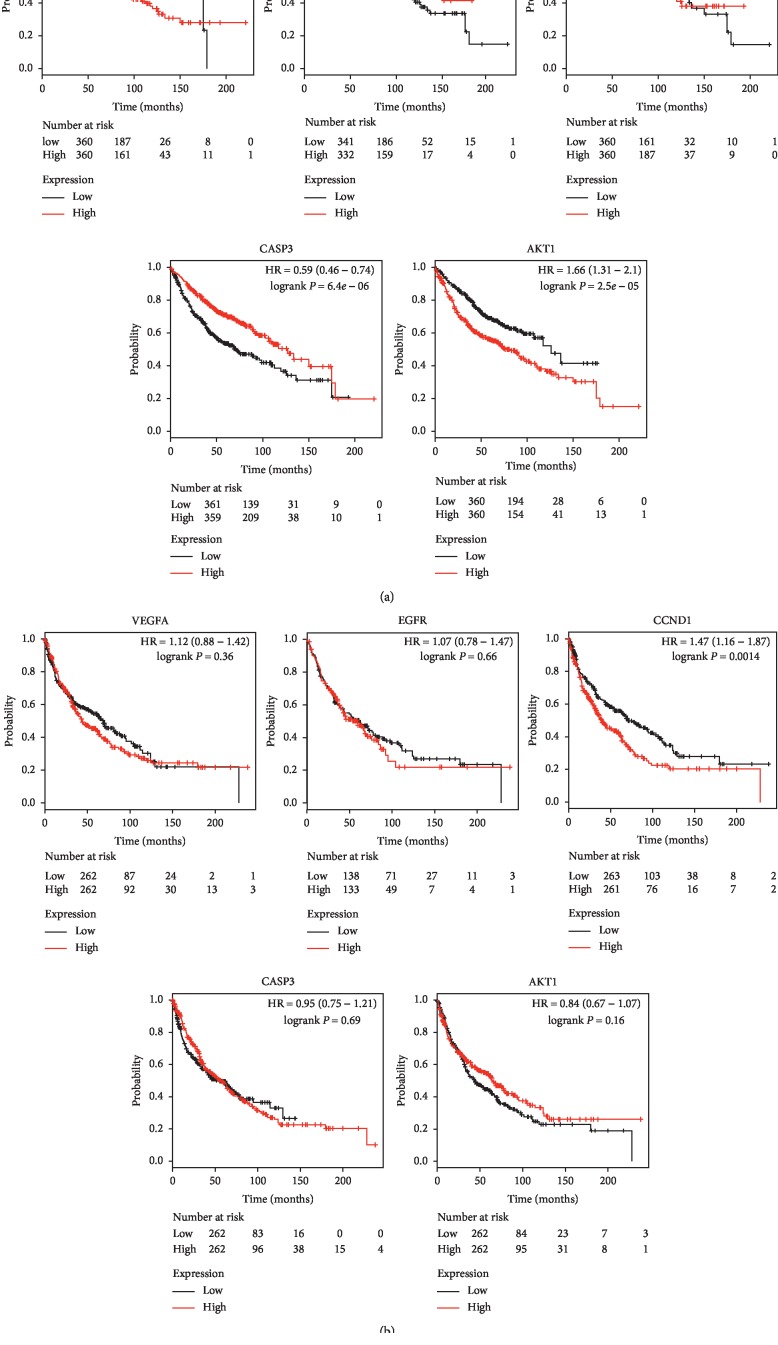
(a) Survival curve analysis of five hub genes and median survival in lung adenocarcinoma patients. (b) Survival curve analysis of five hub genes in patients with lung squamous cell carcinoma. The five hub genes were VEGFA, EGFR, CCND1, CASP3, and AKT1.

We used the Kaplan-Meier Plotter online database to analyze the correlation between five genes with the highest degree (VEGFA, EGFR, CCND1, CASP3, and AKT1) and survival rate by using the Cytoscape software and then drew survival curve. Correlation analysis results showed that the expression of VEGFA, EGFR, CASP3, and AKT1 correlated with the median survival time of lung adenocarcinoma patients (*P* < 0.05). The median survival time of the group with high expression of EGFR and CASP3 was better than that of the group with low expression of EGFR and CASP3, whereas that of the group with high expression of VEGFA and AKT1 was worse than that of the group with low expression of VEGFA and AKT1 (Figure 7(a)). The expression of CCND1 correlated with median survival time in patients with lung squamous cell carcinoma (*P* < 0.05), as shown in Figure 7(b).

## 4. Discussion

Network pharmacology integrates pharmacology, high-throughput sequencing, genomics, and other technologies based on the theory of systems biology, emphasizing multichannel regulation of signaling pathways. In this study, the potential therapeutic target of cinobufacin injection was found by network pharmacology, and the mechanism of action of cinobufacin injection against lung cancer was investigated.

By constructing a network of complex, multicenter genes and gene docking, we determined that the main compounds of cinobufotalin injection were bufalin, cinobufotalin, arenobufagin, and resibufogenin. The potential therapeutic targets of bufalin included MDM2, PIK3CA, MAPK14, and MTOR, which can induce cell death through various mechanisms and exert anticancer properties against lung cancer, liver cancer, and other tumors [23, 24]. Bufalin decreases NCI-H460 cell activity in lung cancer and blocks tumor cells in the G_0_/G_1_ and G_2_/M phases, promoting apoptosis by increasing the production of oxygen-free radicals, caspase-1, and caspase-9 [25, 26]. The researchers established a nude mouse model with xenograft transplantation of A549 tumor and confirmed that cinobufotalin inhibits tumor growth through regulation of the nonapoptotic death pathway of cyclophilin-D in lung cancer cells [27, 28]. Arenobufagin, a cardiac glycoside extracted from toad skin, activates caspase-mediated apoptosis of esophageal cancer cells through exogenous and endogenous pathways [29]. The authors of this previous study have also confirmed that arenobufagin exerts good antivascular effect and blocks the migration and invasion of human umbilical vein endothelial cells by inhibiting the expression of VEGF [30].

In this study, GO and KEGG analyses were carried out on lung-cancer-related genes, potential therapeutic targets of cinobufacin injection, and potential targets of cinobufacin injection for lung cancer. The specific pathogenesis of lung cancer has not been completely defined, and it is related to immune escape, cell proliferation, angiogenesis, and genomic instability and mutation [31]. GO and KEGG analyses of lung-cancer-related genes also showed that activation of the TP53, TNF, and PI3K/Akt/mTOR signaling pathways promotes the occurrence and metastasis of lung cancer. Wang et al. [32] found that the mRNA expression of siglec-15 increases in lung cancer, and siglec-15 continuously inhibits the activity of T lymphocytes. The researchers used mouse tumor models to prove that siglec-15 is a potential candidate for the normalization strategy of tumor immunotherapy. In the potential target enrichment analysis of cinobufacin injection, cinobufacin injection is mainly used to treat lung cancer, liver cancer, bladder cancer, and other malignant tumors. In a clinical study, cinobufacin injection was used to treat 15 cases of stage III and stage IV malignant tumors, such as liver cell cancer and NSCLC. The results showed that tumor size remained unchanged or shrank in six patients [33]. Enrichment analysis of 70 potential targets of cinobufacin injection against lung cancer found that cinobufacin injection exerted clinical efficacy against lung cancer through multiple targets and pathways, involving inhibition of cell proliferation, promotion of apoptosis, antiangiogenesis effect, and immune regulation, which are consistent with the results of studies on lung cancer pathogenesis [34]. In terms of cell cycle and proliferation, the clinical effects of cinobufotalin injection mainly involved the PI3K-Akt (hsa04151), p53 (hsa04115), and MAPK (hsa04010) signaling pathways. The PI3K/AKT/mTOR pathway is a signal transduction pathway involved in various cell function regulation, including cell proliferation, differentiation, migration, and invasion [35]. Cinobufotalin injection blocks cancer cells in the G_2_/M phase, possibly by downregulating the expression of p-Akt, p-mTOR, and p-4E-BP, thus blocking the Akt/mTOR signaling pathway [36]. Wang [37] confirmed that bufalin significantly reduces the incidence of lung metastases and inhibits the epithelial-mesenchymal transformation of tumor cells in orthotopic transplantation and caudate vein injection tumor models. In terms of immune inflammation, cinobufotalin injection plays anti-inflammatory and immunoregulatory roles, mainly, through the TNF (hsa04668), HIF-1 (hsa04066), and Toll-like receptor (hsa04620) signaling pathways. Cinobufotalin injection promotes lymphocyte proliferation, increases macrophage phagocytosis, and enhances immunity by increasing the secretion of IL-2 and IL-10 and regulating the proportion of CD4^+^/CD8^+^ in the spleen [38]. A recent study found that cinobufotalin injection promotes the enrichment of lymphocyte with CD3^+^, CD4^+^, and CD8^+^ in cancer and adjacent tissues and activates the proopiomelanocortin/*β*-endorphin/*μ*-opioid receptor pathway by promoting the proliferation of immune cells to alleviate cancer pain and increase pain threshold in mice [39]. Antiangiogenesis is also an important antitumor mechanism of cinobufotalin injection, which is also reflected in the results of our current study. Yin et al. [40] treated nude mice model of pancreatic cancer with cinobufotalin injection, and the results showed that cinobufotalin injection inhibits the growth of primary pancreatic cancer and hepatic metastases by downregulating the expression of VEGF.

We used protein-protein network interaction to connect potential therapeutic targets of cinobufotalin injection with lung-cancer-related genes, screened hub genes, and carried out survival analysis. The hub genes of potential therapeutic targets of cinobufacin injection, potential therapeutic targets of lung cancer, and potential therapeutic target of cinobufacin injection for lung cancer were identified through the MCODE plugin of the Cytoscape software. We found that the occurrence and metastasis of lung cancer were related to TP53, EGFR, AKT1, MYC, VEGFA, and other genes, consistent with the results of related studies [41]. Cinobufacin injection plays an important role in the treatment of malignant tumors by regulating the expression of AKT1, VEFG, EGFR, CASP3, and CXCL8. In the treatment of lung cancer, cinobufacin injection mainly suppresses cancer cells by regulating key genes of receptor tyrosine kinase-related pathways, such as the VEGFA, EGFR, AKT1, mTOR, and MAPK1 pathways; however, its regulation on CXCL8, which promotes distant metastasis of cancer cells, is weak. Our studies have shown that the hub genes, such as VEGFA, EGFR, CASP3, and AKT1, as potential therapeutic targets of cinobufotalin injection correlated with the survival time of lung adenocarcinoma patients. VEGFA and EGFR are common therapeutic targets for lung cancer and closely related to the survival and prognosis of lung cancer patients [42]. Overactive angiogenesis is a marker of malignant tumors, and VEGFA induces neovascularization and promotes tumor growth and metastasis [43]. The survival time of patients with mutation or high expression of EGFR was significantly prolonged after treatment with small-molecule tyrosine kinase inhibitors [42]. Bufalin downregulates the expression of VEGF in lung cancer, thus inhibiting the proliferation and migration of cancer cells [44, 45]. CASP3, also known as caspase-3, is one of the most important executing factors in the apoptotic pathway, which produces a cascade effect after being activated by its upstream signal and then triggers apoptotic activity [46]. Abnormal expression of this gene is associated with many cancers, such as lung, liver, and intestinal cancers [46–48]. A study has shown that paclitaxel and cisplatin induce apoptosis of lung cancer cells by activating secondary necrosis/pyrophosphorylation mediated by CASP3 and gasdermin E [49]. AKT1 is involved in various biological processes, including cell metabolism, survival, and growth, and its abnormal expression correlated with poor prognosis of tumor patients [50, 51]. Zhu et al. [52] found that bufalin, the main compound of cinobufotalin injection, induces apoptosis of lung cancer cells in a dose- and time-dependent manner by upregulating Bax expression and downregulating BCL-2, thereby blocking the activation of AKT. We found that in lung squamous cell carcinoma, CCND1, the main potential therapeutic target of cinobufotalin injection, was associated with patient survival. CCND1 (located on chromosome 1lql3, approximately 15 kB long), which encodes cyclin D, is a key driving gene for malignant transformation of lung cancer [53]. Baykara et al. [54] detected CCND1 expression level in 85 patients with NSCLC and found that CCND1 was overexpressed in 50 patients (58.8%), 17 of which had lung squamous cell carcinoma.

## 5. Conclusions

We performed pharmacological exploration of cinobufotalin injection and extracted 506 potential therapeutic targets by using network pharmacology and molecular docking. Among the 506 potential therapeutic targets, 70 correlated with the treatment of lung cancer. GO and KEGG enrichment analyses, preliminarily, determined that the mechanism of cinobufotalin injection involved inhibition of cell cycle, promotion of cell apoptosis, regulation of immunity, and antiangiogenesis effect. Moreover, VEGFA, EGFR, CASP3, AKT1, and CCND1 were identified as hub genes in the antilung cancer of cinobufotalin injection. However, further research is needed to verify these results.

## Figures and Tables

**Table 1:** GO analysis of potential target genes of cinobufotalin injection for lung cancer.

Serial number	Term	Count	FDR
GO:0004713	Protein tyrosine kinase activity	15	3.00*E* − 13
GO:0018108	Peptidyl-tyrosine phosphorylation	15	2.41*E* − 12
GO:0042493	Response to drug	18	6.67*E* − 12
GO:0046777	Protein autophosphorylation	15	1.30*E* − 11
GO:0043066	Negative regulation of apoptotic process	20	2.56*E* − 11
GO:0004672	Protein kinase activity	18	7.56*E* − 11
GO:0005524	ATP binding	30	2.21*E* − 10
GO:0004714	Transmembrane receptor protein tyrosine kinase activity	9	3.08*E* − 09
GO:0008284	Positive regulation of cell proliferation	17	7.92*E* − 08
GO:0019899	Enzyme binding	15	8.11*E* − 08
GO:0018105	Peptidyl-serine phosphorylation	11	1.42*E* − 07
GO:0001934	Positive regulation of protein phosphorylation	11	1.66*E* − 07
GO:0005654	Nucleoplasm	34	2.22*E* − 07
GO:0071456	Cellular response to hypoxia	10	3.17*E* − 07
GO:0005829	Cytosol	36	1.09*E* − 06
GO:0070374	Positive regulation of ERK1 and ERK2 cascade	11	3.90*E* − 06
GO:0004674	Protein serine/threonine kinase activity	14	4.59*E* − 06
GO:0016301	Kinase activity	12	4.77*E* − 06
GO:0030335	Positive regulation of cell migration	11	6.33*E* − 06
GO:0006468	Protein phosphorylation	15	6.41*E* − 06

False discovery rate (FDR) corrected *P* < 0.05 was used as an enrichment screening standard. “Count” corresponds to the number of enriched genes in each term. GO: gene ontology.

**Table 2:** KEGG analysis of potential target genes of cinobufotalin injection for lung cancer.

Serial number	Term	Count	FDR
hsa05200	Pathways in cancer	37	2.07*E* − 24
hsa04151	PI3K-Akt signaling pathway	28	3.56*E* − 15
hsa05205	Proteoglycans in cancer	20	3.22*E* − 11
hsa05230	Central carbon metabolism in cancer	13	8.55*E* − 10
hsa04014	Ras signaling pathway	19	3.70*E* − 09
hsa04510	Focal adhesion	18	9.33*E* − 09
hsa04015	Rap1 signaling pathway	18	1.27*E* − 08
hsa05206	MicroRNAs in cancer	20	2.07*E* − 08
hsa04066	HIF-1 signaling pathway	13	1.28*E* − 07
hsa04068	FoxO signaling pathway	14	5.02*E* − 07
hsa05222	Small-cell lung cancer	12	5.63*E* − 07
hsa04914	Progesterone-mediated oocyte maturation	12	7.28*E* − 07
hsa05223	Non-small-cell lung cancer	10	3.37*E* − 06
hsa04668	TNF signaling pathway	12	6.90*E* − 06
hsa04370	VEGF signaling pathway	10	7.43*E* − 06
hsa04012	ErbB signaling pathway	11	1.22*E* − 05
hsa04115	p53 signaling pathway	10	1.75*E* − 05
hsa04917	Prolactin signaling pathway	10	2.95*E* − 05
hsa04919	Thyroid hormone signaling pathway	11	1.82*E* − 04
hsa04915	Estrogen signaling pathway	10	5.51*E* − 04
hsa05231	Choline metabolism in cancer	10	6.55*E* − 04
hsa04114	Oocyte meiosis	10	1.47*E* − 03
hsa04110	Cell cycle	10	3.72*E* − 03
hsa05203	Viral carcinogenesis	12	5.36*E* − 03
hsa04620	Toll-like receptor signaling pathway	9	1.03*E* − 02
hsa04725	Cholinergic synapse	9	1.45*E* − 02
hsa04071	Sphingolipid signaling pathway	9	2.57*E* − 02
hsa04210	Apoptosis	7	3.65*E* − 02
hsa04010	MAPK signaling pathway	12	3.93*E* − 02

False discovery rate (FDR) corrected *P* < 0.05 was used as an enrichment screening standard. “Count” corresponds to the number of enriched genes in each term. KEGG: Kyoto Encyclopedia of Genes and Genomes.

## Data Availability

The data used to support the findings of this study are included within the Supplementary Materials.

## Supplementary Materials

Supplementary Materials Table S1 is about the main effective components of cinobufacin injection. Table S2 is a description of GO analysis results of lung-cancer-related genes. Table S3 is a description of the results of KEGG analysis of lung-cancer-related genes.
