# Supplementary material for: Network Pharmacology Study on the Pharmacological Mechanism of Cinobufotalin Injection against Lung Cancer
Source: Evid Based Complement Alternat Med. 2020 Feb 17;2020:1246742. doi: 10.1155/2020/1246742 (PMC7048923; doi:10.1155/2020/1246742)
Supplement: Supplementary Materials — Table S1 is about the main effective components of cinobufacin injection. Table S2 is a description of GO analysis results of lung-cancer-related genes. Table S3 is a description of the results of KEGG analysis of lung-cancer-related genes. [file 1246742.f1.zip › 1246742.f1/Table S1.doc]

**Table S1: The components of cinobufotalin injection.**

| Number | Compound | Molecular Formula | Chemical Structure |
| --- | --- | --- | --- |
| 1 | Xanthine | C_5_H_4_N_4_O_2_ |  |
| 2 | Adenine | C_5_H_5_N_5_ |  |
| 3 | Adenosine | C_10_H_13_N_5_O_4_ | <http://tcmspw.com/tcmspmol/MOL006946.mol2> |
| 4 | Arenobufagin | C_24_H_32_O_6_ |  |
| 5 | Bufalin | C_24_H_34_O_4_ |  |
| 6 | Bufarenogin | C24H32O6 |  |
| 7 | Bufotalin | C26H36O6 |  |
| 8 | Bufotenidine | C_13_H_18_N_2_O |  |
| 9 | Bufotenine | C_12_H_16_N_2_O |  |
| 10 | Bufothionine | C12H15N2O3S+ |  |
| 11 | Cinobufagin | C26H34O6 |  |
| 12 | Cinobufotalin | C_26_H_34_O_7_ |  |
| 13 | Dehydrobufotenine | C_12_H_15_N_2_O^+^ |  |
| 14 | Gamabufotalin | C_24_H_34_O_5_ |  |
| 15 | Guanosine | C_10_H_13_N_5_O_5_ |  |
| 16 | Hypoxanthine | C_5_H_4_N_4_O |  |
| 17 | Marinobufagin | C_24_H_32_O_5_ |  |
| 18 | Resibufogenin | C24H32O4 |  |
| 19 | Serotonin | C_10_H_12_N_2_O |  |
| 20 | Telocinobufagin | C24H34O5 |  |
| 21 | Thymine | C_5_H_6_N_2_O_2_ |  |
| 22 | Uracil | C_4_H_4_N_2_O_2_ |  |
| 23 | Uridine | C_9_H_12_N_2_O_6_ |  |
