# Supplementary material for: Network Pharmacology Study on the Pharmacological Mechanism of Cinobufotalin Injection against Lung Cancer
Source: Evid Based Complement Alternat Med. 2020 Feb 17;2020:1246742. doi: 10.1155/2020/1246742 (PMC7048923; doi:10.1155/2020/1246742)
Supplement: Supplementary Materials — Table S1 is about the main effective components of cinobufacin injection. Table S2 is a description of GO analysis results of lung-cancer-related genes. Table S3 is a description of the results of KEGG analysis of lung-cancer-related genes. [file 1246742.f1.zip › 1246742.f1/Table S2.doc]

**Table S2: The GO analysis of the lung cancer-related genes**

| **Serial number** | **Term** | **Count** | **FDR** |
| --- | --- | --- | --- |
| GO:0042493 | response to drug | 63 | 2.73E-39 |
| GO:0043066 | negative regulation of apoptotic process | 73 | 2.44E-38 |
| GO:0005515 | protein binding | 308 | 3.58E-36 |
| GO:0045893 | positive regulation of transcription, DNA-templated | 72 | 1.12E-33 |
| GO:0008285 | negative regulation of cell proliferation | 63 | 3.36E-32 |
| GO:0008284 | positive regulation of cell proliferation | 67 | 9.23E-32 |
| GO:0005615 | extracellular space | 105 | 2.25E-31 |
| GO:0045944 | positive regulation of transcription from RNA polymerase II promoter | 91 | 1.43E-29 |
| GO:0010628 | positive regulation of gene expression | 47 | 2.41E-25 |
| GO:0042802 | identical protein binding | 72 | 1.42E-23 |
| GO:0000165 | MAPK cascade | 45 | 2.69E-23 |
| GO:0019899 | enzyme binding | 49 | 7.87E-23 |
| GO:0030335 | positive regulation of cell migration | 37 | 5.48E-21 |
| GO:0070374 | positive regulation of ERK1 and ERK2 cascade | 36 | 1.12E-20 |
| GO:0018108 | peptidyl-tyrosine phosphorylation | 34 | 1.80E-20 |
| GO:0004713 | protein tyrosine kinase activity | 32 | 2.29E-20 |
| GO:0001666 | response to hypoxia | 35 | 7.55E-20 |
| GO:0005576 | extracellular region | 97 | 5.86E-20 |
| GO:0005654 | nucleoplasm | 130 | 1.74E-18 |
| GO:0043065 | positive regulation of apoptotic process | 42 | 4.79E-18 |
| GO:0008083 | growth factor activity | 32 | 1.20E-17 |
| GO:0048015 | phosphatidylinositol-mediated signaling | 27 | 3.44E-17 |
| GO:0007050 | cell cycle arrest | 30 | 4.08E-17 |
| GO:0006974 | cellular response to DNA damage stimulus | 35 | 4.45E-17 |
| GO:0048661 | positive regulation of smooth muscle cell proliferation | 22 | 4.49E-17 |
| GO:0014066 | regulation of phosphatidylinositol 3-kinase signaling | 24 | 5.87E-17 |
| GO:0008630 | intrinsic apoptotic signaling pathway in response to DNA damage | 20 | 1.16E-16 |
| GO:0050731 | positive regulation of peptidyl-tyrosine phosphorylation | 24 | 2.08E-16 |
| GO:0000732 | strand displacement | 16 | 9.78E-16 |
| GO:0046934 | phosphatidylinositol-4,5-bisphosphate 3-kinase activity | 21 | 1.67E-15 |
| GO:0042060 | wound healing | 23 | 2.11E-15 |
| GO:0048146 | positive regulation of fibroblast proliferation | 20 | 2.63E-15 |
| GO:0032355 | response to estradiol | 24 | 2.74E-15 |
| GO:0043410 | positive regulation of MAPK cascade | 23 | 2.84E-15 |
| GO:0005088 | Ras guanyl-nucleotide exchange factor activity | 26 | 3.01E-15 |
| GO:0007568 | aging | 30 | 3.85E-15 |
| GO:0001934 | positive regulation of protein phosphorylation | 27 | 4.46E-15 |
| GO:0000731 | DNA synthesis involved in DNA repair | 17 | 9.07E-15 |
| GO:0005737 | cytoplasm | 183 | 9.52E-15 |
| GO:0000122 | negative regulation of transcription from RNA polymerase II promoter | 58 | 2.18E-14 |
| GO:0006915 | apoptotic process | 51 | 3.38E-14 |
| GO:0004714 | transmembrane receptor protein tyrosine kinase activity | 17 | 3.40E-14 |
| GO:0008283 | cell proliferation | 41 | 4.78E-14 |
| GO:0071260 | cellular response to mechanical stimulus | 21 | 4.78E-14 |
| GO:0045471 | response to ethanol | 24 | 8.56E-14 |
| GO:0014068 | positive regulation of phosphatidylinositol 3-kinase signaling | 20 | 2.11E-13 |
| GO:0009636 | response to toxic substance | 22 | 2.11E-13 |
| GO:0006955 | immune response | 43 | 2.11E-13 |
| GO:0043406 | positive regulation of MAP kinase activity | 19 | 4.11E-13 |
| GO:0001938 | positive regulation of endothelial cell proliferation | 20 | 4.11E-13 |
| GO:0009986 | cell surface | 46 | 9.44E-13 |
| GO:0046777 | protein autophosphorylation | 28 | 1.24E-12 |
| GO:0007165 | signal transduction | 72 | 2.07E-12 |
| GO:0016301 | kinase activity | 32 | 1.71E-12 |
| GO:0001525 | angiogenesis | 31 | 2.28E-12 |
| GO:0005524 | ATP binding | 83 | 2.04E-12 |
| GO:0010629 | negative regulation of gene expression | 25 | 4.13E-12 |
| GO:0005634 | nucleus | 181 | 4.71E-12 |
| GO:0071407 | cellular response to organic cyclic compound | 18 | 8.46E-12 |
| GO:0045766 | positive regulation of angiogenesis | 23 | 9.29E-12 |
| GO:0045740 | positive regulation of DNA replication | 16 | 9.29E-12 |
| GO:0050679 | positive regulation of epithelial cell proliferation | 18 | 1.16E-11 |
| GO:0045429 | positive regulation of nitric oxide biosynthetic process | 16 | 1.40E-11 |
| GO:0005125 | cytokine activity | 27 | 1.37E-11 |
| GO:0046854 | phosphatidylinositol phosphorylation | 21 | 1.92E-11 |
| GO:0042981 | regulation of apoptotic process | 29 | 3.82E-11 |
| GO:0009897 | external side of plasma membrane | 28 | 3.55E-11 |
| GO:0010332 | response to gamma radiation | 14 | 5.26E-11 |
| GO:0071222 | cellular response to lipopolysaccharide | 22 | 7.16E-11 |
| GO:0016032 | viral process | 33 | 1.78E-10 |
| GO:0042803 | protein homodimerization activity | 52 | 1.52E-10 |
| GO:0008134 | transcription factor binding | 32 | 1.73E-10 |
| GO:0000187 | activation of MAPK activity | 21 | 2.62E-10 |
| GO:0031093 | platelet alpha granule lumen | 16 | 2.21E-10 |
| GO:0050918 | positive chemotaxis | 14 | 3.49E-10 |
| GO:0003682 | chromatin binding | 37 | 3.10E-10 |
| GO:0010165 | response to X-ray | 12 | 4.67E-10 |
| GO:0006281 | DNA repair | 29 | 4.67E-10 |
| GO:0034644 | cellular response to UV | 15 | 4.86E-10 |
| GO:0097421 | liver regeneration | 13 | 6.43E-10 |
| GO:0001701 | in utero embryonic development | 26 | 6.75E-10 |
| GO:0005829 | cytosol | 125 | 5.57E-10 |
| GO:0030324 | lung development | 18 | 8.25E-10 |
| GO:0038128 | ERBB2 signaling pathway | 14 | 1.21E-09 |
| GO:0006468 | protein phosphorylation | 39 | 2.23E-09 |
| GO:0031663 | lipopolysaccharide-mediated signaling pathway | 13 | 2.64E-09 |
| GO:0005886 | plasma membrane | 143 | 2.74E-09 |
| GO:0042127 | regulation of cell proliferation | 25 | 3.93E-09 |
| GO:1902042 | negative regulation of extrinsic apoptotic signaling pathway via death domain receptors | 13 | 4.07E-09 |
| GO:0004672 | protein kinase activity | 34 | 3.49E-09 |
| GO:0006977 | DNA damage response, signal transduction by p53 class mediator resulting in cell cycle arrest | 16 | 5.88E-09 |
| GO:1902895 | positive regulation of pri-miRNA transcription from RNA polymerase II promoter | 11 | 5.90E-09 |
| GO:0097192 | extrinsic apoptotic signaling pathway in absence of ligand | 13 | 6.16E-09 |
| GO:0043234 | protein complex | 35 | 4.76E-09 |
| GO:0003684 | damaged DNA binding | 16 | 5.58E-09 |
| GO:0042056 | chemoattractant activity | 12 | 5.93E-09 |
| GO:0044212 | transcription regulatory region DNA binding | 26 | 9.01E-09 |
| GO:0048471 | perinuclear region of cytoplasm | 43 | 8.79E-09 |
| GO:0043627 | response to estrogen | 16 | 1.23E-08 |
| GO:0002576 | platelet degranulation | 19 | 1.47E-08 |
| GO:0019901 | protein kinase binding | 34 | 1.23E-08 |
| GO:0043235 | receptor complex | 20 | 1.37E-08 |
| GO:0051091 | positive regulation of sequence-specific DNA binding transcription factor activity | 19 | 2.06E-08 |
| GO:0007507 | heart development | 24 | 2.23E-08 |
| GO:0031100 | organ regeneration | 14 | 2.62E-08 |
| GO:0045892 | negative regulation of transcription, DNA-templated | 39 | 3.40E-08 |
| GO:2000811 | negative regulation of anoikis | 10 | 3.81E-08 |
| GO:0051726 | regulation of cell cycle | 20 | 4.26E-08 |
| GO:1901796 | regulation of signal transduction by p53 class mediator | 20 | 4.26E-08 |
| GO:0071456 | cellular response to hypoxia | 18 | 4.53E-08 |
| GO:0051897 | positive regulation of protein kinase B signaling | 17 | 5.55E-08 |
| GO:0002020 | protease binding | 18 | 7.68E-08 |
| GO:0043491 | protein kinase B signaling | 12 | 1.03E-07 |
| GO:0000790 | nuclear chromatin | 23 | 7.86E-08 |
| GO:0097191 | extrinsic apoptotic signaling pathway | 13 | 1.06E-07 |
| GO:0043524 | negative regulation of neuron apoptotic process | 20 | 1.31E-07 |
| GO:0060020 | Bergmann glial cell differentiation | 8 | 1.40E-07 |
| GO:0043525 | positive regulation of neuron apoptotic process | 13 | 1.45E-07 |
| GO:0048538 | thymus development | 13 | 1.45E-07 |
| GO:0002053 | positive regulation of mesenchymal cell proliferation | 11 | 1.51E-07 |
| GO:0007179 | transforming growth factor beta receptor signaling pathway | 17 | 2.34E-07 |
| GO:0000784 | nuclear chromosome, telomeric region | 19 | 1.82E-07 |
| GO:0046982 | protein heterodimerization activity | 36 | 2.06E-07 |
| GO:0007173 | epidermal growth factor receptor signaling pathway | 14 | 2.95E-07 |
| GO:0003690 | double-stranded DNA binding | 16 | 2.62E-07 |
| GO:0043547 | positive regulation of GTPase activity | 40 | 3.43E-07 |
| GO:2000352 | negative regulation of endothelial cell apoptotic process | 11 | 3.59E-07 |
| GO:0051781 | positive regulation of cell division | 13 | 4.55E-07 |
| GO:0007265 | Ras protein signal transduction | 15 | 4.89E-07 |
| GO:0001657 | ureteric bud development | 12 | 5.83E-07 |
| GO:0010468 | regulation of gene expression | 17 | 8.53E-07 |
| GO:0032496 | response to lipopolysaccharide | 21 | 8.64E-07 |
| GO:0043552 | positive regulation of phosphatidylinositol 3-kinase activity | 11 | 1.14E-06 |
| GO:0019903 | protein phosphatase binding | 14 | 1.07E-06 |
| GO:0032403 | protein complex binding | 23 | 1.08E-06 |
| GO:0046677 | response to antibiotic | 11 | 1.63E-06 |
| GO:0001541 | ovarian follicle development | 12 | 1.92E-06 |
| GO:0006954 | inflammatory response | 31 | 2.02E-06 |
| GO:0045121 | membrane raft | 22 | 1.73E-06 |
| GO:0010718 | positive regulation of epithelial to mesenchymal transition | 11 | 2.30E-06 |
| GO:0030878 | thyroid gland development | 10 | 2.74E-06 |
| GO:0090399 | replicative senescence | 8 | 2.90E-06 |
| GO:0045840 | positive regulation of mitotic nuclear division | 10 | 4.12E-06 |
| GO:0043536 | positive regulation of blood vessel endothelial cell migration | 9 | 5.26E-06 |
| GO:0097296 | activation of cysteine-type endopeptidase activity involved in apoptotic signaling pathway | 8 | 6.17E-06 |
| GO:0016020 | membrane | 85 | 5.24E-06 |
| GO:2001240 | negative regulation of extrinsic apoptotic signaling pathway in absence of ligand | 11 | 8.00E-06 |
| GO:0048010 | vascular endothelial growth factor receptor signaling pathway | 14 | 8.10E-06 |
| GO:0045669 | positive regulation of osteoblast differentiation | 13 | 9.46E-06 |
| GO:0000082 | G1/S transition of mitotic cell cycle | 16 | 1.00E-05 |
| GO:0005901 | caveola | 13 | 8.12E-06 |
| GO:0000724 | double-strand break repair via homologous recombination | 14 | 1.15E-05 |
| GO:0070062 | extracellular exosome | 100 | 9.97E-06 |
| GO:0005925 | focal adhesion | 29 | 1.01E-05 |
| GO:0006260 | DNA replication | 19 | 1.43E-05 |
| GO:0010863 | positive regulation of phospholipase C activity | 7 | 1.50E-05 |
| GO:0022617 | extracellular matrix disassembly | 14 | 1.61E-05 |
| GO:0050900 | leukocyte migration | 17 | 1.69E-05 |
| GO:2000379 | positive regulation of reactive oxygen species metabolic process | 10 | 1.75E-05 |
| GO:0042542 | response to hydrogen peroxide | 12 | 1.79E-05 |
| GO:0030198 | extracellular matrix organization | 21 | 1.99E-05 |
| GO:0060045 | positive regulation of cardiac muscle cell proliferation | 9 | 2.10E-05 |
| GO:1904707 | positive regulation of vascular smooth muscle cell proliferation | 8 | 2.23E-05 |
| GO:0031295 | T cell costimulation | 14 | 2.24E-05 |
| GO:0018105 | peptidyl-serine phosphorylation | 17 | 2.41E-05 |
| GO:0043200 | response to amino acid | 10 | 2.41E-05 |
| GO:0051384 | response to glucocorticoid | 13 | 2.47E-05 |
| GO:0005102 | receptor binding | 28 | 2.06E-05 |
| GO:0005667 | transcription factor complex | 20 | 1.91E-05 |
| GO:0008584 | male gonad development | 15 | 2.75E-05 |
| GO:0001658 | branching involved in ureteric bud morphogenesis | 11 | 3.07E-05 |
| GO:0048754 | branching morphogenesis of an epithelial tube | 9 | 3.16E-05 |
| GO:0016303 | 1-phosphatidylinositol-3-kinase activity | 11 | 3.01E-05 |
| GO:0006978 | DNA damage response, signal transduction by p53 class mediator resulting in transcription of p21 class mediator | 8 | 3.89E-05 |
| GO:0044130 | negative regulation of growth of symbiont in host | 8 | 3.89E-05 |
| GO:0006919 | activation of cysteine-type endopeptidase activity involved in apoptotic process | 14 | 4.87E-05 |
| GO:0016324 | apical plasma membrane | 24 | 3.80E-05 |
| GO:0050680 | negative regulation of epithelial cell proliferation | 12 | 5.08E-05 |
| GO:0060440 | trachea formation | 6 | 5.17E-05 |
| GO:0033138 | positive regulation of peptidyl-serine phosphorylation | 13 | 5.91E-05 |
| GO:0070301 | cellular response to hydrogen peroxide | 12 | 6.17E-05 |
| GO:0060749 | mammary gland alveolus development | 8 | 6.49E-05 |
| GO:0006298 | mismatch repair | 10 | 7.82E-05 |
| GO:0000723 | telomere maintenance | 10 | 1.02E-04 |
| GO:0006935 | chemotaxis | 16 | 1.19E-04 |
| GO:0036092 | phosphatidylinositol-3-phosphate biosynthetic process | 11 | 1.50E-04 |
| GO:0008022 | protein C-terminus binding | 19 | 1.29E-04 |
| GO:0008625 | extrinsic apoptotic signaling pathway via death domain receptors | 10 | 1.71E-04 |
| GO:0009887 | organ morphogenesis | 14 | 1.72E-04 |
| GO:0045931 | positive regulation of mitotic cell cycle | 9 | 1.82E-04 |
| GO:2000145 | regulation of cell motility | 9 | 1.82E-04 |
| GO:0009611 | response to wounding | 12 | 1.83E-04 |
| GO:0006979 | response to oxidative stress | 15 | 2.12E-04 |
| GO:0006953 | acute-phase response | 10 | 2.17E-04 |
| GO:0001948 | glycoprotein binding | 12 | 1.96E-04 |
| GO:0017015 | regulation of transforming growth factor beta receptor signaling pathway | 8 | 2.44E-04 |
| GO:0071375 | cellular response to peptide hormone stimulus | 8 | 2.44E-04 |
| GO:0060391 | positive regulation of SMAD protein import into nucleus | 7 | 2.84E-04 |
| GO:0007169 | transmembrane receptor protein tyrosine kinase signaling pathway | 14 | 2.88E-04 |
| GO:0047485 | protein N-terminus binding | 14 | 2.46E-04 |
| GO:0002039 | p53 binding | 12 | 2.70E-04 |
| GO:0007131 | reciprocal meiotic recombination | 9 | 3.31E-04 |
| GO:0007267 | cell-cell signaling | 22 | 3.33E-04 |
| GO:0031954 | positive regulation of protein autophosphorylation | 8 | 3.60E-04 |
| GO:0071480 | cellular response to gamma radiation | 8 | 3.60E-04 |
| GO:0030168 | platelet activation | 15 | 3.72E-04 |
| GO:0003677 | DNA binding | 69 | 3.11E-04 |
| GO:0051092 | positive regulation of NF-kappaB transcription factor activity | 16 | 3.78E-04 |
| GO:0048701 | embryonic cranial skeleton morphogenesis | 9 | 4.37E-04 |
| GO:0000400 | four-way junction DNA binding | 7 | 3.85E-04 |
| GO:0035690 | cellular response to drug | 12 | 4.83E-04 |
| GO:0005739 | mitochondrion | 56 | 3.71E-04 |
| GO:2001241 | positive regulation of extrinsic apoptotic signaling pathway in absence of ligand | 7 | 4.88E-04 |
| GO:0046427 | positive regulation of JAK-STAT cascade | 8 | 5.18E-04 |
| GO:0031625 | ubiquitin protein ligase binding | 23 | 4.53E-04 |
| GO:0000722 | telomere maintenance via recombination | 9 | 5.72E-04 |
| GO:0060333 | interferon-gamma-mediated signaling pathway | 12 | 6.53E-04 |
| GO:0097190 | apoptotic signaling pathway | 12 | 6.53E-04 |
| GO:0071347 | cellular response to interleukin-1 | 12 | 6.53E-04 |
| GO:0016342 | catenin complex | 6 | 5.38E-04 |
| GO:0007411 | axon guidance | 17 | 7.23E-04 |
| GO:0000729 | DNA double-strand break processing | 7 | 7.98E-04 |
| GO:0050729 | positive regulation of inflammatory response | 12 | 8.72E-04 |
| GO:0009612 | response to mechanical stimulus | 11 | 9.43E-04 |
| GO:0005657 | replication fork | 7 | 9.29E-04 |
| GO:0007569 | cell aging | 8 | 0.001380313 |
| GO:0016323 | basolateral plasma membrane | 17 | 0.001086402 |
| GO:0042523 | positive regulation of tyrosine phosphorylation of Stat5 protein | 7 | 0.001902162 |
| GO:0048568 | embryonic organ development | 7 | 0.001902162 |
| GO:0004716 | receptor signaling protein tyrosine kinase activity | 6 | 0.001607177 |
| GO:0045080 | positive regulation of chemokine biosynthetic process | 6 | 0.002022334 |
| GO:2001237 | negative regulation of extrinsic apoptotic signaling pathway | 9 | 0.002373377 |
| GO:0008217 | regulation of blood pressure | 11 | 0.002392881 |
| GO:0060326 | cell chemotaxis | 11 | 0.002392881 |
| GO:0014065 | phosphatidylinositol 3-kinase signaling | 8 | 0.002456092 |
| GO:0031069 | hair follicle morphogenesis | 8 | 0.002456092 |
| GO:0008013 | beta-catenin binding | 12 | 0.00220551 |
| GO:0006302 | double-strand break repair | 11 | 0.002766309 |
| GO:0020037 | heme binding | 15 | 0.002421853 |
| GO:0034097 | response to cytokine | 10 | 0.002942488 |
| GO:0050821 | protein stabilization | 15 | 0.002944789 |
| GO:0043388 | positive regulation of DNA binding | 8 | 0.003213979 |
| GO:0010033 | response to organic substance | 8 | 0.003213979 |
| GO:0044267 | cellular protein metabolic process | 14 | 0.003227565 |
| GO:0070411 | I-SMAD binding | 6 | 0.002894362 |
| GO:1901215 | negative regulation of neuron death | 9 | 0.00359335 |
| GO:0038083 | peptidyl-tyrosine autophosphorylation | 9 | 0.00359335 |
| GO:0008201 | heparin binding | 16 | 0.00307892 |
| GO:0005578 | proteinaceous extracellular matrix | 20 | 0.003214536 |
| GO:0030308 | negative regulation of cell growth | 14 | 0.004293497 |
| GO:0004674 | protein serine/threonine kinase activity | 25 | 0.003580366 |
| GO:0006928 | movement of cell or subcellular component | 12 | 0.004660806 |
| GO:0001822 | kidney development | 12 | 0.004660806 |
| GO:0042475 | odontogenesis of dentin-containing tooth | 10 | 0.004801549 |
| GO:0005178 | integrin binding | 13 | 0.004175376 |
| GO:0045599 | negative regulation of fat cell differentiation | 9 | 0.005309648 |
| GO:0046326 | positive regulation of glucose import | 8 | 0.005322757 |
| GO:0030332 | cyclin binding | 7 | 0.004449781 |
| GO:0001570 | vasculogenesis | 10 | 0.005612545 |
| GO:0014911 | positive regulation of smooth muscle cell migration | 7 | 0.005636877 |
| GO:0002052 | positive regulation of neuroblast proliferation | 7 | 0.005636877 |
| GO:0032405 | MutLalpha complex binding | 5 | 0.004738041 |
| GO:0060664 | epithelial cell proliferation involved in salivary gland morphogenesis | 5 | 0.005916712 |
| GO:0006959 | humoral immune response | 10 | 0.006538573 |
| GO:0042771 | intrinsic apoptotic signaling pathway in response to DNA damage by p53 class mediator | 8 | 0.006747644 |
| GO:1900182 | positive regulation of protein localization to nucleus | 7 | 0.007747586 |
| GO:0004003 | ATP-dependent DNA helicase activity | 8 | 0.006649131 |
| GO:0008094 | DNA-dependent ATPase activity | 8 | 0.006649131 |
| GO:0050852 | T cell receptor signaling pathway | 15 | 0.008057787 |
| GO:0001974 | blood vessel remodeling | 8 | 0.00847674 |
| GO:0051216 | cartilage development | 10 | 0.008789811 |
| GO:0071356 | cellular response to tumor necrosis factor | 13 | 0.008964614 |
| GO:0007623 | circadian rhythm | 11 | 0.00913771 |
| GO:0003697 | single-stranded DNA binding | 12 | 0.007771912 |
| GO:0005518 | collagen binding | 10 | 0.007862504 |
| GO:2000573 | positive regulation of DNA biosynthetic process | 6 | 0.009785613 |
| GO:0060644 | mammary gland epithelial cell differentiation | 6 | 0.009785613 |
| GO:0051000 | positive regulation of nitric-oxide synthase activity | 7 | 0.010458528 |
| GO:0010634 | positive regulation of epithelial cell migration | 8 | 0.010559362 |
| GO:0000186 | activation of MAPKK activity | 9 | 0.010881046 |
| GO:0010595 | positive regulation of endothelial cell migration | 9 | 0.010881046 |
| GO:0032729 | positive regulation of interferon-gamma production | 9 | 0.010881046 |
| GO:0032869 | cellular response to insulin stimulus | 11 | 0.011635686 |
| GO:0032760 | positive regulation of tumor necrosis factor production | 9 | 0.012866919 |
| GO:0007585 | respiratory gaseous exchange | 8 | 0.013050361 |
| GO:0001837 | epithelial to mesenchymal transition | 8 | 0.013050361 |
| GO:0051593 | response to folic acid | 5 | 0.013567998 |
| GO:0035924 | cellular response to vascular endothelial growth factor stimulus | 7 | 0.013891604 |
| GO:0003148 | outflow tract septum morphogenesis | 7 | 0.013891604 |
| GO:0007219 | Notch signaling pathway | 13 | 0.014259727 |
| GO:0045334 | clathrin-coated endocytic vesicle | 6 | 0.011594888 |
| GO:0000307 | cyclin-dependent protein kinase holoenzyme complex | 6 | 0.011594888 |
| GO:0048565 | digestive tract development | 8 | 0.016010451 |
| GO:0071560 | cellular response to transforming growth factor beta stimulus | 9 | 0.017766955 |
| GO:0031012 | extracellular matrix | 20 | 0.013573064 |
| GO:0070371 | ERK1 and ERK2 cascade | 7 | 0.018184243 |
| GO:0010507 | negative regulation of autophagy | 8 | 0.019506542 |
| GO:0048469 | cell maturation | 8 | 0.019506542 |
| GO:0042599 | lamellar body | 5 | 0.015028888 |
| GO:0005159 | insulin-like growth factor receptor binding | 6 | 0.017517643 |
| GO:0005161 | platelet-derived growth factor receptor binding | 6 | 0.017517643 |
| GO:0022408 | negative regulation of cell-cell adhesion | 6 | 0.022026261 |
| GO:0050930 | induction of positive chemotaxis | 6 | 0.022026261 |
| GO:0048714 | positive regulation of oligodendrocyte differentiation | 6 | 0.022026261 |
| GO:0042593 | glucose homeostasis | 12 | 0.022645137 |
| GO:0007596 | blood coagulation | 16 | 0.022878321 |
| GO:0060070 | canonical Wnt signaling pathway | 11 | 0.022984754 |
| GO:0006310 | DNA recombination | 11 | 0.022984754 |
| GO:0048589 | developmental growth | 7 | 0.023490192 |
| GO:2000134 | negative regulation of G1/S transition of mitotic cell cycle | 7 | 0.023490192 |
| GO:0042130 | negative regulation of T cell proliferation | 8 | 0.023612069 |
| GO:0010888 | negative regulation of lipid storage | 5 | 0.026668484 |
| GO:0070723 | response to cholesterol | 5 | 0.026668484 |
| GO:0010544 | negative regulation of platelet activation | 5 | 0.026668484 |
| GO:0001102 | RNA polymerase II activating transcription factor binding | 8 | 0.02230325 |
| GO:0018107 | peptidyl-threonine phosphorylation | 8 | 0.028407312 |
| GO:0045930 | negative regulation of mitotic cell cycle | 7 | 0.029980258 |
| GO:0050999 | regulation of nitric-oxide synthase activity | 7 | 0.029980258 |
| GO:0035162 | embryonic hemopoiesis | 6 | 0.031466976 |
| GO:0035994 | response to muscle stretch | 6 | 0.031466976 |
| GO:0043154 | negative regulation of cysteine-type endopeptidase activity involved in apoptotic process | 10 | 0.032744627 |
| GO:0000079 | regulation of cyclin-dependent protein serine/threonine kinase activity | 8 | 0.033979712 |
| GO:0045668 | negative regulation of osteoblast differentiation | 8 | 0.033979712 |
| GO:0010575 | positive regulation of vascular endothelial growth factor production | 7 | 0.037842989 |
| GO:0051262 | protein tetramerization | 8 | 0.040424181 |
| GO:0055093 | response to hyperoxia | 6 | 0.043783207 |
| GO:0034713 | type I transforming growth factor beta receptor binding | 5 | 0.037794782 |
| GO:0032930 | positive regulation of superoxide anion generation | 5 | 0.047175164 |
| GO:0008635 | activation of cysteine-type endopeptidase activity involved in apoptotic process by cytochrome c | 5 | 0.047175164 |
| GO:0060068 | vagina development | 5 | 0.047175164 |
| GO:0032689 | negative regulation of interferon-gamma production | 7 | 0.047285295 |
| GO:0035116 | embryonic hindlimb morphogenesis | 7 | 0.047285295 |
| GO:0021983 | pituitary gland development | 7 | 0.047285295 |
| GO:0042733 | embryonic digit morphogenesis | 9 | 0.049015592 |
| False discovery rate (FDR) corrected *P*<0.05 was used as an enrichment screening standard. ‘Count’ corresponds to the number of enriched genes in each term. GO, Gene Ontology | | | |
