# Supplementary material for: Network Pharmacology Study on the Pharmacological Mechanism of Cinobufotalin Injection against Lung Cancer
Source: Evid Based Complement Alternat Med. 2020 Feb 17;2020:1246742. doi: 10.1155/2020/1246742 (PMC7048923; doi:10.1155/2020/1246742)
Supplement: Supplementary Materials — Table S1 is about the main effective components of cinobufacin injection. Table S2 is a description of GO analysis results of lung-cancer-related genes. Table S3 is a description of the results of KEGG analysis of lung-cancer-related genes. [file 1246742.f1.zip › 1246742.f1/Table S3.doc]

**Table 3S: The KEGG analysis of the lung cancer-related genes**

| **Serial number** | **Term** | **Count** | **FDR** |
| --- | --- | --- | --- |
| hsa05206 | MicroRNAs in cancer | 109 | 3.05E-64 |
| hsa05200 | Pathways in cancer | 124 | 2.55E-63 |
| hsa04151 | PI3K-Akt signaling pathway | 79 | 8.33E-27 |
| hsa05205 | Proteoglycans in cancer | 58 | 3.21E-24 |
| hsa04068 | FoxO signaling pathway | 45 | 6.91E-21 |
| hsa04066 | HIF-1 signaling pathway | 38 | 5.26E-20 |
| hsa05223 | Non-small cell lung cancer | 30 | 1.08E-19 |
| hsa05222 | Small cell lung cancer | 34 | 9.94E-18 |
| hsa04115 | p53 signaling pathway | 28 | 1.23E-14 |
| hsa05230 | Central carbon metabolism in cancer | 27 | 3.99E-14 |
| hsa04668 | TNF signaling pathway | 33 | 2.78E-13 |
| hsa04012 | ErbB signaling pathway | 30 | 2.78E-13 |
| hsa04510 | Focal adhesion | 45 | 8.44E-13 |
| hsa04014 | Ras signaling pathway | 46 | 5.94E-12 |
| hsa04210 | Apoptosis | 24 | 3.19E-11 |
| hsa04015 | Rap1 signaling pathway | 43 | 4.49E-11 |
| hsa04917 | Prolactin signaling pathway | 24 | 9.10E-10 |
| hsa04919 | Thyroid hormone signaling pathway | 30 | 1.14E-09 |
| hsa04110 | Cell cycle | 31 | 1.47E-09 |
| hsa04010 | MAPK signaling pathway | 43 | 3.05E-08 |
| hsa05203 | Viral carcinogenesis | 37 | 1.78E-07 |
| hsa04370 | VEGF signaling pathway | 20 | 2.08E-07 |
| hsa04550 | Signaling pathways regulating pluripotency of stem cells | 30 | 2.10E-07 |
| hsa04620 | Toll-like receptor signaling pathway | 24 | 6.81E-06 |
| hsa05231 | Choline metabolism in cancer | 23 | 1.37E-05 |
| hsa04722 | Neurotrophin signaling pathway | 25 | 1.75E-05 |
| hsa04914 | Progesterone-mediated oocyte maturation | 21 | 2.45E-05 |
| hsa04915 | Estrogen signaling pathway | 22 | 4.89E-05 |
| hsa05202 | Transcriptional misregulation in cancer | 29 | 6.24E-05 |
| hsa04664 | Fc epsilon RI signaling pathway | 18 | 7.74E-05 |
| hsa04071 | Sphingolipid signaling pathway | 24 | 8.15E-05 |
| hsa04920 | Adipocytokine signaling pathway | 18 | 1.23E-04 |
| hsa04520 | Adherens junction | 18 | 1.54E-04 |
| hsa04150 | mTOR signaling pathway | 16 | 2.90E-04 |
| hsa04350 | TGF-beta signaling pathway | 19 | 3.98E-04 |
| hsa04810 | Regulation of actin cytoskeleton | 31 | 7.94E-04 |
| hsa04621 | NOD-like receptor signaling pathway | 15 | 0.001160079 |
| hsa04390 | Hippo signaling pathway | 25 | 0.001594337 |
| hsa04062 | Chemokine signaling pathway | 28 | 0.002151272 |
| hsa04630 | Jak-STAT signaling pathway | 24 | 0.002765144 |
| hsa04662 | B cell receptor signaling pathway | 16 | 0.003220862 |
| hsa04064 | NF-kappa B signaling pathway | 18 | 0.00333544 |
| hsa04060 | Cytokine-cytokine receptor interaction | 32 | 0.006056823 |
| hsa04380 | Osteoclast differentiation | 22 | 0.006490422 |
| hsa04650 | Natural killer cell mediated cytotoxicity | 21 | 0.007696138 |
| hsa03440 | Homologous recombination | 10 | 0.015957352 |
| hsa04152 | AMPK signaling pathway | 20 | 0.031562225 |
| False discovery rate (FDR) corrected *P*<0.05 was used as an enrichment screening standard. ‘Count’ corresponds to the number of enriched genes in each term. KEGG, Kyoto Encyclopedia of Genes and Genome. | | | |
